# Physiology of the Nitrite-Oxidizing Bacterium *Candidatus* Nitrotoga sp. CP45 Enriched From a Colorado River

**DOI:** 10.3389/fmicb.2021.709371

**Published:** 2021-08-16

**Authors:** Munira A. Lantz, Andrew M. Boddicker, Michael P. Kain, Owen M. C. Berg, Courtney D. Wham, Annika C. Mosier

**Affiliations:** Department of Integrative Biology, University of Colorado Denver, Denver, CO, United States

**Keywords:** nitrite-oxidizing bacteria, freshwater, nitrification, water quality, nitrotoga, antibiotics, physiology, temperature

## Abstract

Nitrogen cycling microbes, including nitrite-oxidizing bacteria (NOB), perform critical ecosystem functions that help mitigate anthropogenic stresses and maintain ecosystem health. Activity of these beneficial nitrogen cycling microbes is dictated in part by the microorganisms’ response to physicochemical conditions, such as temperature, pH, and nutrient availability. NOB from the newly described *Candidatus* Nitrotoga genus have been detected in a wide range of habitats across the globe, yet only a few organisms within the genus have been physiologically characterized. For freshwater systems where NOB are critical for supporting aquatic life, *Ca.* Nitrotoga have been previously detected but little is known about the physiological potential of these organisms or their response to changing environmental conditions. Here, we determined functional response to environmental change for a representative freshwater species of *Ca.* Nitrotoga (*Ca*. Nitrotoga sp. CP45, enriched from a Colorado river). The physiological findings demonstrated that CP45 maintained nitrite oxidation at pH levels of 5–8, at temperatures from 4 to 28°C, and when incubated in the dark. Light exposure and elevated temperature (30°C) completely halted nitrite oxidation. *Ca*. Nitrotoga sp. CP45 maintained nitrite oxidation upon exposure to four different antibiotics, and potential rates of nitrite oxidation by river sediment communities were also resilient to antibiotic stress. We explored the *Ca*. Nitrotoga sp. CP45 genome to make predictions about adaptations to enable survival under specific conditions. Overall, these results contribute to our understanding of the versatility of a representative freshwater *Ca*. Nitrotoga sp. Identifying the specific environmental conditions that maximize NOB metabolic rates may ultimately direct future management decisions aimed at restoring impacted systems.

## Introduction

Nitrite-oxidizing bacteria (NOB) play fundamental roles in maintaining the health and resilience of freshwater habitats by regulating nitrogen transformations. NOB are responsible for the second step of nitrification (oxidizing nitrite to nitrate) and have three key impacts on aquatic habitats. First, they are responsible for the formation of nitrate, which provides a critical nitrogen source for microbial and plant assimilation. Second, they provide a substrate for denitrification, which results in the formation of gaseous dinitrogen for evolution out of the aquatic habitat. Third, they reduce nitrite toxicity to fish and other aquatic organisms (and indirectly reduce ammonia toxicity by consuming the end-product of microbial ammonia oxidation). If nitrite oxidation rates are reduced or halted due to environmental perturbation, nitrite concentrations will increase and negative effects, such as toxicity, hypoxia, and loss of biodiversity, may propagate through the ecosystem.

*Nitrobacter* and *Nitrospira* are often identified as the primary NOB present in freshwater systems (Hovanec et al., 1998; Féray et al., 1999; Altmann et al., 2003; Cébron and Garnier, 2005). Sequences from the recently discovered *Candidatus* Nitrotoga (Alawi et al., 2007) have now also been reported in a wide range of freshwater habitats, including a subglacial lake (Christner et al., 2014), wastewater treatment plants (WWTPs)/activated sludge (Alawi et al., 2009; Lücker et al., 2014; Kitzinger et al., 2018; Wegen et al., 2019), a groundwater cave system (Chen et al., 2009), biologically active filter for drinking water (White et al., 2012), the tidal reach area of a freshwater river (Fan et al., 2016), a freshwater aquaculture plant (Hüpeden et al., 2016), and rivers (Li et al., 2011; Boddicker and Mosier, 2018). *Ca.* Nitrotoga-like sequences range in relative abundance from ∼0.01 to 10% of the total bacterial community (which is similar to estimates for other NOB) across globally distributed freshwater habitats (Boddicker and Mosier, 2018). The relationship between *Ca.* Nitrotoga relative abundance, nitrite oxidation rates, and *in situ* nitrite concentrations remains understudied.

Despite the detection of *Ca.* Nitrotoga gene sequences in a variety of freshwater habitats, little is known about the physiological potential of these organisms or their response to changing environmental conditions. Across the entire genus, physiology data have only been reported for a small number of *Ca.* Nitrotoga cultures, which were enriched from permafrost, activated sludge, an aquaculture system, and coastal sand (Alawi et al., 2007, 2009; Hüpeden et al., 2016; Ishii et al., 2017, 2020; Kitzinger et al., 2018; Wegen et al., 2019). These physiological studies have indicated that optimal temperatures for *Ca.* Nitrotoga physiological activity range from 10° to 28°C and that *Ca.* Nitrotoga has been cultured at pH values ranging from 6.8 to 8.3. There are no prior reports on the physiology of freshwater *Ca.* Nitrotoga so it is unknown whether or not these NOB have similar activity to *Ca.* Nitrotoga enriched from other habitats.

Here, we determine the physiology of a newly identified *Ca.* Nitrotoga species from a natural freshwater system, *Ca*. Nitrotoga sp. CP45 (Boddicker and Mosier, 2018). In this study, we measure nitrite oxidation under varying conditions of light, pH, temperature, and antibiotic exposure to better characterize and predict environmental parameters that may lead to physiological activity/inactivity within freshwater systems. We explore the CP45 genome to make predictions about which genes may be involved in the physiological responses that were observed. Physiology and genomic potential of *Ca*. Nitrotoga sp. CP45 is directly relevant to Colorado ecosystems, but can also be viewed as more broadly applicable to other freshwater systems since CP45 is closely related to other freshwater *Ca*. Nitrotoga sp. found across the globe (based on prior gene sequence analyses; Boddicker and Mosier, 2018). Determining the physiological limits of the newly described *Ca*. Nitrotoga sp. can improve our understanding of how these bacteria respond under natural and stressed conditions in the environment.

## Materials and Methods

### Cultivation and Genomic Sequencing of *Ca*. Nitrotoga sp. CP45

*Ca*. Nitrotoga sp. CP45 was previously enriched from water column samples collected from the Cache la Poudre River near Greeley, CO (site CP45; latitude 40.41774, longitude −104.64017). Briefly, cultures were grown in Freshwater Nitrite Oxidizer Medium (FNOM) (Boddicker and Mosier, 2018) with 0.3 mM nitrite and incubated at room temperature (∼23°C) in the dark. The cultures were enriched with NOB from the *Ca.* Nitrotoga genus (based on PCR and amplicon sequencing; *Ca.* Nitrotoga was 16–24% enriched at the time of genomic sequencing, and approximately 65% enriched upon further purification) (Boddicker and Mosier, 2018). The *Ca*. Nitrotoga sp. CP45 genome was previously sequenced and annotated (Boddicker and Mosier, 2018). Genome sequence and phylogenetic analyses showed that the NOB in the culture represented a new species within the *Ca.* Nitrotoga genus (Boddicker and Mosier, 2018). Here, further annotation of genes specific to processes described in this study was conducted using the eggNOG database and mapper (Huerta-Cepas et al., 2017, 2019), BacMet2 (Pal et al., 2014), virulence factor database (VFDB; Liu et al., 2019), Interpro (Blum et al., 2021), and BLAST (Altschul et al., 1990).

### Measurement of Nitrite Concentrations

NOB activity in culture is commonly measured by following rates of substrate (nitrite) utilization or product (nitrate) formation (Prosser, 1989). Here, cultures were regularly monitored for nitrite utilization using a Griess nitrite color reagent composed of 10 g sulfanilamide (SULF), 1.0 g N-(1-napthyl) ethylenediamine dihydrochloride (NEDD), 100 ml 85% phosphoric acid, and MilliQ water to a final volume of 1 L (Griess-Romijn van Eck, 1966; American Public Health Association, American Water Works Association, and Water Environment Federation, 2017). The solution was stored in the dark at 4°C for up to 1 month in an amber bottle wrapped in aluminum foil. Griess nitrite color reagent was mixed with culture samples at a 1:1 ratio. Nitrite concentrations were determined by quantitative spectrophotometric measurements at 543 nm according to *Standard Methods*, method 4,500-NO${}_{2}^{-}$ B Colorimetric Method (American Public Health Association, American Water Works Association, and Water Environment Federation, 2017) using a BioTek Synergy HT plate reader (BioTek, Winooski, VT). Pseudoreplicates (× 2) were measured for each individual nitrite sample. Nitrite concentrations present in sampled media were calculated using resultant end point absorbance readings and associated nitrite standards (0–0.3 mM NO_2_^–^) prepared in sterile FNOM. In all, more than 10,000 individual nitrite measurements were made for the experiments described below.

### Growth Curve

Four large batches of culture (700 ml FNOM with 7 ml *Ca*. Nitrotoga sp. CP45 inoculum) were incubated at 23°C and grown over the course of 5 days. Nitrite was measured from subsamples collected approximately every 6 h. At select time points, 10 ml of each culture was filtered through 0.2-μm Supor 200 filters (Pall, New York, NY) and immediately frozen at −80°C. DNA was extracted as previously described (Boddicker and Mosier, 2018).

Nitrite concentrations were measured as described above. Nitrate + nitrite (NOx) concentrations were determined using Vanadium (III) Chloride in hydrochloric acid combined with the Griess reagents (SULF and NEDD) (Miranda et al., 2001). Following color development, absorbance was read as described above with pseudoreplicates (× 2) for each sample. NOx concentrations were calculated using resultant end point absorbance readings and associated standards (0–0.3 mM NO${}_{3}^{\text{-}}$) prepared in sterile FNOM. Nitrate was determined by subtracting nitrite values from the NOx values.

Extracted DNA was amplified in triplicate quantitative PCR (qPCR) reactions using *Ca.* Nitrotoga 16S rRNA gene-specific primers NTG200F and NTG840R (Alawi et al., 2007). Reactions were carried out using a StepOnePlus Real-Time PCR System (Thermo Fisher Scientific, Waltham, MA). Each 20-μl reaction included 10 μl of FailSafe Premix E (Lucigen, Madison, WI) [with 20X SYBR Green Nucleic Acid Dye (Invitrogen, Carlsbad, CA) and 0.2% Tween-20], 5.95 μl of nuclease-free water, 0.4 μl of 25 μM ROX Passive Reference Dye (Invitrogen, Carlsbad, CA), 1.6 μl of 25 mM MgCl_2_, 0.4 μl of 10 μM forward and reverse primers, 0.05 μl of 50 mg/ml BSA, and 0.2 μl of AmpliTaq polymerase 5 U/μl (Invitrogen, Carlsbad, CA). Standard curves (10^3^–10^8^ copies/μl) were generated using purified PCR product from the *Ca.* Nitrotoga 16S rRNA primers on the CP45 enrichment culture. The qPCR was run with three technical replicates from each time point. Cycling conditions were 96°C for 2 min followed by 40 cycles of 96°C for 50 s, 58°C for 50 s, 72°C for 50 s, and 84°C for 10 s to acquire an image. *R*^2^ values for the standard curves were > 0.995 for all runs. qPCR efficiencies ranged from 68.3 to 69.2%. Melt curve analysis was performed after each run with plate reads at a temperature increment of 0.3°C.

### Physiology Experiments

All physiology treatments utilized FNOM with a final concentration of 0.3 mM nitrite distributed in sterile 20-ml borosilicate glass scintillation vials (Thermo Fisher Scientific, Waltham, MA). Prior to use, all scintillation vials were soaked in 1.9% HCl for a minimum of 24 h, followed by triplicate rinsing with DI water, triplicate rinsing with Milli-Q water, drying, and autoclaving at 15 psi and 250°F for 60 min followed immediately by a 30-min drying exhaustive cycle. The inoculum culture was regularly monitored for nitrite oxidation activity. Immediately after depletion of nitrite, *Ca*. Nitrotoga sp. CP45 was inoculated into FNOM at a ratio of 100 μl culture to 10 ml of FNOM in scintillation vials. Triplicate cultures were inoculated for each experimental condition and grown under aerobic conditions. Each experiment (temperature, pH, light/dark, and antibiotics) was conducted at separate times, so the starting inoculum may vary slightly from experiment to experiment (e.g., physiological state and cell numbers). Therefore, data should be compared within an experiment, but not necessarily between experiments (e.g., comparisons among pH 5 and pH 7, but not pH 5 versus light).

Effects of light on Nitrotoga nitrite oxidation rates were tested with triplicate vials being exposed to continuous direct light with an illumination of 486 μEinsteins m^–2^s^–1^, while a separate triplicate vial set was wrapped in aluminum foil. Temperature range was tested in triplicate vials housed in incubators at the following temperatures: 4, 10, 15, 20, 23, 25, 28, and 30°C. pH treatments were performed using FNOM adjusted to pH 5.0, pH 5.7, pH 6.0, pH 7.0, and pH 8.0 using either 10% (3.26 M) hydrochloric acid (HCl, 22° Bé) or 1 M sodium hydroxide (NaOH). For antibiotic treatments, solutions of erythromycin, penicillin (penicillin G), sulfamethoxazole, and trimethoprim were made at concentrations of 5, 50, and 500 nM in a base of FNOM. All vials (with the exception of the temperature experiment) were incubated at 23°C. All vials were incubated in the dark (with the exception of the light treatment).

Nitrite concentrations were determined in 100-μl subsamples from each vial collected at approximately 6-h intervals (as described above). Nitrite oxidation rates were calculated across three time points within the period of logarithmic nitrite consumption for each vial sampled. Individual rates were calculated for each replicate and then averaged.

### River Mesocosm Experiments

Three sampling sites were selected along the South Platte River Basin to evaluate nitrite oxidation capabilities of the endogenous microbial communities upon exposure to antibiotics. Each site was chosen due to contrasting landscape use and water chemistry (Storteboom et al., 2010a,b): (1) site SPCC along the South Platte River upstream of Clear Creek (−104.949010, 39.827140) representative of WWTP effluent-dominated water chemistry; (2) site SPKER along South Platte River at Colorado Highway 37 near Kersey (−104.563270, 40.412250) representative of a mixed WWTP effluent and animal feeding operation influenced water chemistry; and (3) CP45 at Cache la Poudre at County Road 45 in Greeley (−104.640170, 40.417740) dominated by animal feeding operations.

Surface sediment samples were obtained from each site and immediately placed on ice for processing at the lab within a few hours. Three sediment samples from each location were physically homogenized and 1.5 g (wet weight) was placed in a sterile scintillation vial with 15 ml of 0.3 mM nitrite FNOM plus 500 nM of an antibiotic (penicillin, erythromycin, sulfamethoxazole, or trimethoprim). Antibiotics were chosen based on their presence in river waterways as well as their antibacterial properties and pharmacokinetics. Biological controls contained river sediment and FNOM with no antibiotics. Blank controls contained FNOM and sediment samples (1.5 g) that were sterilized by autoclaving on a dry cycle at 15 psi at a constant temperature of 250°C for 30 min, followed by cooling and vortexing for 30 s prior to a second dry autoclaving run.

Water column mesocosm experiments were set up utilizing the same three predetermined sites. For each site, 250 ml of surface water from three separate grab-bottle captures were placed into a sterile, acid-washed Nalgene and mixed for homogenization. Homogenized site water was amended with nitrite (0.3 mM final concentration) and one of the antibiotics at 500 nM final concentration (penicillin, erythromycin, sulfamethoxazole, or trimethoprim). Biological controls contained river water amended with nitrite, but with no antibiotics. Blank control water samples (45 ml) were sterilized by first filtering the sample with a 0.45-μm 25-mm PES Nalgene syringe filter, then double filtering using 0.2-μm 25-mm PES Nalgene syringe filters.

For sediment and water column mesocosm experiments, triplicates of each treatment vial were incubated at 23°C in the dark. Nitrite concentrations and nitrite oxidation potential rates were determined as described above. DNA was extracted from sediment and water column inoculum for each site in duplicate, and NOB were quantified by qPCR as described above using primers targeting *Ca.* Nitrotoga (NTG200F 5′-CTCGCGTTTTCGGAGCGG and NTG840R 5′-CTAAGGAAGTCTCCTCCC) (Alawi et al., 2007) and *Nitrospira* (Nspra675F 5′-GCGGTGAAATGCGTAGAKATCG and Nspra746R 5′-TCAGCGTCAGRWAYGTTCCAGAG) (Graham et al., 2007).

### Environmental Parameters

Environmental parameters were measured at the river site where the *Ca*. Nitrotoga sp. CP45 culture inoculum was collected (see above). Briefly, surface water parameters were measured approximately every 4–8 weeks from May 2015 to July 2016. Temperature and pH were measured with a YSI Professional Plus handheld multiparameter meter (YSI Incorporated, Yellow Springs, Ohio).

Water quality data were obtained from the Northern Water Conservancy District (Northern Water Conservancy District, 2016) at a site 2 miles downstream from the CP45 sampling site (latitude 40.4244, longitude −104.6000). Data included water temperature, pH, turbidity, and dissolved inorganic nitrogen concentrations. Samples were obtained monthly from April of 2015 through November of 2016 (with the exception of January and December), analyzed at a USGS certified laboratory, and approved in a QA/QC process. At a sampling site 9 miles upstream from CP45 (latitude 40.5013, longitude −104.9673), water temperature was obtained at 15-min intervals around-the-clock from April of 2015 through May of 2016 using a HOBO ProV2 temperature logger (Onset Computer Corporation, Bourne, MA).

## Results and Discussion

### Study Site Description

We previously enriched *Ca*. Nitrotoga sp. CP45 from the Cache la Poudre River water column in the South Platte River Basin in Colorado (Boddicker and Mosier, 2018). Genomic sequencing revealed the metabolic potential to couple the oxidation of nitrite with aerobic respiration for energy conservation *via* a novel nitrite oxidoreductase enzyme (Boddicker and Mosier, 2018). *Ca*. Nitrotoga sp. CP45 16S rRNA gene sequences were highly conserved with other *Ca.* Nitrotoga-like sequences found in freshwater habitats across the globe (Boddicker and Mosier, 2018).

As a representative freshwater *Ca*. Nitrotoga sp., here we extend this prior genomic sequencing work to evaluate the ability of *Ca*. Nitrotoga sp. CP45 to oxidize nitrite under a range of chemical and physical conditions to better understand how these NOB might respond in varying habitats across the river system. The South Platte River flows across the Colorado Front Range where multiple land-use types result in fluctuating contaminant inputs, including urban WWTP discharge, agricultural land-use areas with concentrated fertilizer and pesticide application, and land areas with a high density of animal feed operations (Storteboom et al., 2010a,b). Pristine headwaters in the Rocky Mountains near central Colorado migrate through a gradient of human activities, from urban sites in the Denver metropolitan area, to mixed setting sites dominated by urban and agricultural land use, to the most northern sites dominated by predominantly agricultural land use (Dennehy et al., 1998).

### *Ca.* Nitrotoga sp. CP45 Growth and Nitrite Oxidation

**FIGURE 1:**
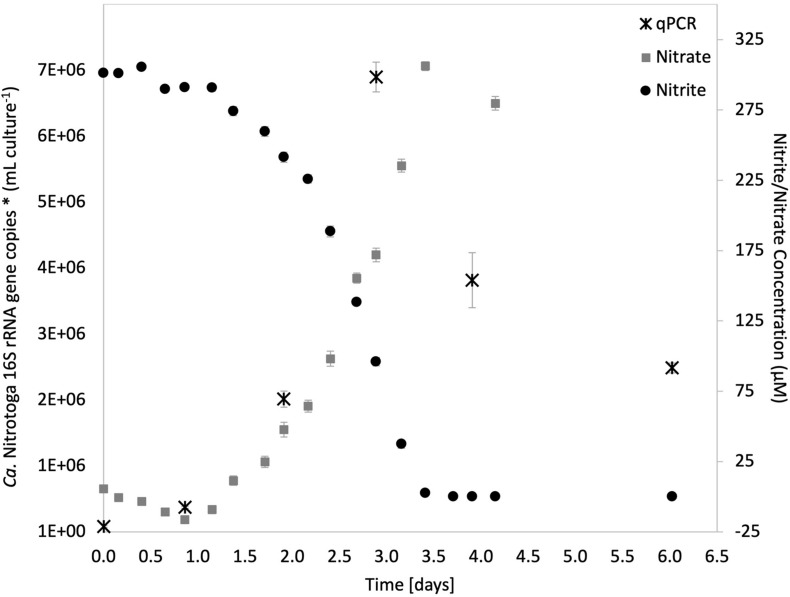
Nitrite oxidation in the *Ca*. Nitrotoga sp. CP45 enrichment culture coupled with increased concentrations of nitrate and copies of *Ca.* Nitrotoga 16S rRNA genes. Nitrite (black circles) and nitrate (gray squares) values are averages of quadruplicate vials (each with duplicate sample measurements) at each sampling time point with error bars representing ± the standard error of the mean (SEM). Gene copy numbers measured by qPCR (black stars) are averages ± SEM error bars across three technical replicates. If not visible, error bars are smaller than data points.

Batch cultures of *Ca*. Nitrotoga sp. CP45 grown in media with 0.3 mM nitrite (NO${}_{2}^{-}$) revealed that nitrite consumption corresponded with increased nitrate concentrations (at near-stoichiometric levels) and increased 16S rRNA gene copy numbers (utilized as a proxy for cell growth) (Figure 1). Maximum 16S rRNA gene copies at 69 h post-inoculation coincided with a logarithmic decline in nitrite concentrations, followed by a tapering off once nitrite was depleted (Figure 1). Accounting for genomic estimates showing that the *C*a. Nitrotoga sp. CP45 genome had two copies of the 16S rRNA gene per cell (Boddicker and Mosier, 2018), qPCR estimates suggest that CP45 reached a maximum of 3.5 × 10^6^ cells per ml culture. Mean nitrite oxidation rate (calculated across four measurements during logarithmic nitrite oxidation) averaged 167 ± 7 μM NO${}_{2}^{-}$ day^−1^ (Figure 1). *Ca*. Nitrotoga sp. CP45 oxidized NO${}_{2}^{-}$ at concentrations ranging from 0.15 to 4.5 mM NO${}_{2}^{-}$ (rates not determined).

Though some NOB are inhibited by ammonia (e.g., *Nitrospira* inhibited at 0.04–0.08 mg NH_3_-N L^–1^ produced by the addition of ammonium in the media; Blackburne et al., 2007), recent studies reveal that other NOB benefit from the addition of ammonium for both growth and stable nitrite oxidation (Sorokin et al., 2012; Ishii et al., 2017, 2020; Spieck et al., 2019; Wegen et al., 2019). Specifically, some species of *Ca.* Nitrotoga have demonstrated stimulated nitrite oxidation with the addition of 0.1–30 mM ammonium (Ishii et al., 2017, 2020; Wegen et al., 2019). Here, *Ca*. Nitrotoga sp. CP45 oxidized 0.3 mM NO${}_{2}^{\text{-}}$ in the presence of 0.15–4.5 mM ammonium (rates not determined). These concentrations were higher than *in situ* river water ammonium concentrations at the CP45 sampling site (Supplementary Figure 1), suggesting that water column ammonium does not interfere with the potential for riverine nitrite oxidation. Prior genomic analyses indicated that CP45 encodes an ammonium transporter (*amtB*) potentially facilitating ammonium assimilation (Boddicker and Mosier, 2018). Further research should evaluate how inorganic nitrogen concentrations impact the ecology of *Ca*. Nitrotoga sp. CP45, including resource partitioning with other co-occurring NOB depending on enzyme kinetics and the range of substrate concentrations.

### Impact of Light Exposure on *Ca.* Nitrotoga sp. CP45 Nitrite Oxidation

**FIGURE 2:**
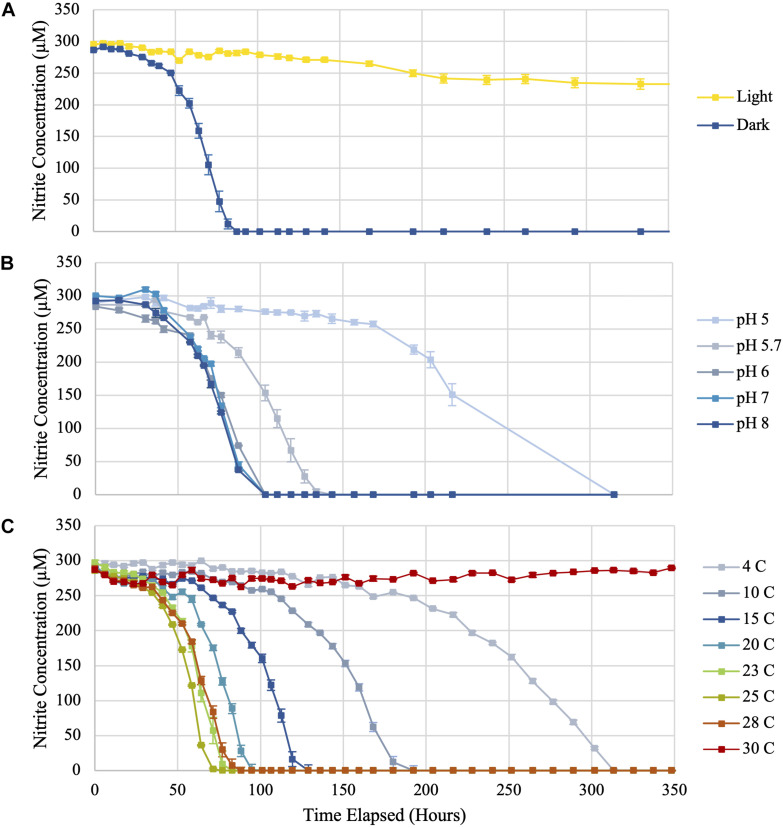
Nitrite utilization by *Ca*. Nitrotoga sp. CP45 when **(A)** exposed to continuous light compared to incubation in darkness, **(B)** grown in media at varying pH values, and **(C)** incubated at eight different temperatures (4, 10, 15, 20, 23, 25, 28, and 30°C). Values plotted are averages of triplicate vials at each sampling time point with error bars representing ± the standard error of the mean (SEM). If not visible, error bars are smaller than data points.

Microorganisms in aquatic environments are exposed to light at varying intensities given fluctuating depths and water transparency; however, no prior studies have evaluated the effect of light exposure on *Ca.* Nitrotoga. To test photosensitivity, *Ca*. Nitrotoga sp. CP45 was incubated under continuous illumination at 486 μEinsteins m^–2^ s^–1^ while separate biological replicates were maintained in darkness. *Ca*. Nitrotoga sp. CP45 exhibited no nitrite oxidation when grown in continuous illumination, compared to cultures incubated in the dark that oxidized nitrite at a rate of 194 μM day^–1^ (Figure 2 and Supplementary Table 1). Photoeffect on *Ca*. Nitrotoga sp. CP45 could be the result of photooxidation of *c-*type cytochromes, previously reported in *Ca.* Nitrotoga genomes (Boddicker and Mosier, 2018; Kitzinger et al., 2018; Ishii et al., 2020). Cytochrome c has been shown to absorb light in the visible spectrum causing photodynamic destruction of amino acid residues (Spikes and Livingston, 1969), resulting in light-induced cell death (Bock, 1970; Bock and Wagner, 2013). In some habitats, photochemical instability of nitrite (Zafiriou and True, 1979) could result in photoinhibition by decreasing substrate availability, though that is unlikely in the experiments conducted here since nitrite concentrations were constant over time in the CP45 incubations and in the controls that contained no biomass.

Photoinhibitory effects have been found to occur in several other nitrifying microbes (*Nitrosomonas*, *Nitrosococcus*, *Nitrospira*, *Nitrococcus*, and *Nitrobacter* species) (Hooper and Terry, 1974; Yoshioka and Saijo, 1984; Vanzella et al., 1989; Guerrero and Jones, 1996; Kaplan et al., 2000; Merbt et al., 2012). Photoinhibition documented in *Ca*. Nitrotoga sp. CP45 may also impact other *Ca*. Nitrotoga sp. with similar cell structures and metabolisms. This trait likely impacts the environmental range of these organisms, with nitrite oxidation activity occurring primarily in habitats devoid of light. *Ca*. Nitrotoga sp. CP45 was enriched from a river water column that exhibits daily and seasonal fluctuations in light exposure in part dependent on the levels of suspended particulate and organic matter. Turbidity (as a measure of water clarity) near the CP45 river site ranged from 4 to 118 NTU over time (Supplementary Figure 2), which spans the range of values typically seen in pristine streams and heavily sedimented rivers. High water column turbidity levels may alleviate some of the photoinhibition for nitrite oxidation by *Ca*. Nitrotoga sp. CP45 within the water column. However, turbidity could have secondary effects on NOB related to increased water temperature as the suspended particles absorb more heat or reduce dissolved oxygen concentrations from decreased photosynthesis (Austin et al., 2017). *Ca*. Nitrotoga sp. likely also benefit from photoprotection within riverbed sediments.

### Impact of Varying pH on *Ca*. Nitrotoga sp. CP45 Nitrite Oxidation

**FIGURE 3:**
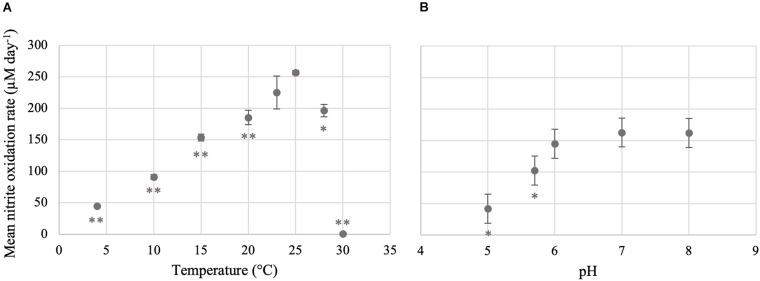
Temperature and pH optima for the nitrite-oxidizing activity of *Ca*. Nitrotoga sp. CP45. **(A)** Mean nitrite oxidation rates during incubation at varying temperatures. **(B)** Mean nitrite oxidation rates during incubation at varying pH conditions. Values are means of triplicate biological replicates with ± standard deviations (error bars). If not visible, error bars are smaller than data points. Values that are significantly different from the optima (highest rate; 25°C for temperature and pH 7 for pH) by *t*-test are indicated by asterisks as follows: *p < 0.05; **p < 0.01.

Nitrite was oxidized in *Ca*. Nitrotoga sp. CP45 cultures at pH 5.0–8.0 (Figures 2, 3 and Supplementary Table 1). The highest rates of nitrite oxidation occurred at pH 7 and pH 8 (162 μM day^–1^), with a slightly lower rate at pH 6. At the most acidic conditions tested (pH 5.0), mean nitrite oxidation rates were 42 μM day^–1^. These results were consistent with prior *Ca.* Nitrotoga physiology studies demonstrating near-neutral pH culture conditions and optima: *Ca.* Nitrotoga arctica cultivated at pH 7.4–7.6 (optima not determined), *Ca*. Nitrotoga sp. AM1 cultivated at pH of 8.0–8.3 (optima at pH 8.3), *Ca*. Nitrotoga sp. HW29 cultivated at pH 6.8–7.4 (optima at pH 6.8), *Ca*. Nitrotoga sp. HAM-1 cultivated at pH 7.4–7.6 (optima not determined), and *Ca*. Nitrotoga sp. fabula cultivated at pH 6.6–8.0 (optimum 7.1–7.6) (Alawi et al., 2007, 2009; Hüpeden et al., 2016; Ishii et al., 2017; Kitzinger et al., 2018). At the CP45 river enrichment site, the water pH ranged from 7.8 to 8.1 over a 10-month time period (Supplementary Figure 2), so these physiology tests suggest that *Ca*. Nitrotoga sp. CP45 is likely able to perform nitrite oxidation at all pH levels measured within the river year-round. It cannot be ruled out that pH changed over the course of the incubation; however, the media has some buffering capacity and NOB cultures typically become slightly more acidic over time due to proton production during nitrite oxidation (Prosser, 1989). Other community members in the enrichment culture may have impacted these results (see below); however, no other NOB were identified in the 16S rRNA gene or genome sequence dataset and no other electron donors or accepters were added to the media.

Decreased nitrite oxidation in *Ca*. Nitrotoga sp. CP45 cultures at decreased pH values is likely the result of a combination of factors associated with abiotic chemistry and bacterial physiology. pH value has a direct impact on the equilibrium between nitrite (NO_2_^–^) and nitrous acid (HNO_2_) and therefore directly affects the availability of nitrite in a system. According to the nitrous acid equilibrium, the concentration of nitrous acid increases as pH decreases, resulting in a corresponding decrease in the nitrite pool available for NOB energy generation (Philips et al., 2002). Nitrite oxidation has been shown to be inhibited at low pH due to the toxicity of nitrous acid (Prosser, 1989 and references therein). Additionally, acidic pH conditions can be toxic to NOB cells by causing a disruption in the proton concentration that is intricately involved in cellular bioenergetics through the proton motive force (Krulwich et al., 2011). Decreased pH has also been found to alter the catalytic activities of enzymes involved in nitrogen transformations by compromising their structural integrity (Schreiber et al., 2012; Blum et al., 2018). In non-neutral pH conditions, additional energy must be expended to combat pH stress, such as the expression of membrane pumps to actively uptake protons or the efflux of protons to maintain internal pH. Collectively, low nitrite pools and physiological stress at low pH can potentially lead to insufficient energy for growth (Prosser, 1989).

Nitrite oxidation at pH 5.0 suggests that *Ca*. Nitrotoga sp. CP45 may be acid tolerant, which has been shown for other NOB such as *Nitrospira* sp. (Takahashi et al., 2020) and *Nitrobacter* sp. IOacid (Hankinson and Schmidt, 1988) enriched from acid soils. We probed the *Ca*. Nitrotoga sp. CP45 genome for genes predicted to be involved in acid homeostasis. We identified a putative ActS/PrrB/RegB family redox-sensitive histidine kinase and an acid tolerance regulatory protein (ActR), which may be involved in the ActS/ActR two-component system that plays an important role in acid tolerance and oxidant resistance (Tiwari et al., 1996; Tang et al., 2017). Another potential mechanism of acid tolerance is to regulate proton and cation transport in order to reduce the overall influx of protons into the cytoplasm under low pH conditions (Booth, 1985; Baker-Austin and Dopson, 2007). The CP45 genome encoded a large number of inorganic ion transport proteins, including several cation:proton antiporters, cation-transporting ATPases (P-type), and cation-related signal transduction proteins. The *Ca*. Nitrotoga sp. CP45 genome contained many genes for cell wall/cell membrane biosynthesis and DNA repair, which have been proposed as mechanisms of pH tolerance (Baker-Austin and Dopson, 2007 and references within), though further research is necessary to determine if these genes are used in structural modifications for pH homeostasis. Similar to the acidophilic ammonia-oxidizing archaeon, *Ca.* Nitrosotalea devanaterra (Lehtovirta-Morley et al., 2016), the *Ca*. Nitrotoga sp. CP45 genome encoded two carbonic anhydrases that may have a dual function for carbon cycling and cytoplasmic buffering to prevent acidification (Sachs et al., 2005). Some of these CP45 proteins are also found in other *Ca.* Nitrotoga genomes, which suggests that the proteins may not necessarily be involved in acid tolerance or that the other *Ca.* Nitrotoga strains may be capable of nitrite oxidation at lower pH conditions.

Since *Ca*. Nitrotoga sp. CP45 appeared to oxidize nitrite across all tested pH conditions (5.0–8.0), it would be useful to further probe survival and metabolism at pH conditions below pH 5.0 and above pH 8.0, which would be a considerable advantage in engineered systems such as WWTPs that rely on NOB function. Prior studies of nitrite oxidation in WWTP have shown that NOB (e.g., *Nitrobacter* and *Nitrospira*) operate optimally within a near-neutral range and exhibit reduced activity or cessation of nitrite oxidation at acidic conditions (Grunditz and Dalhammar, 2001; Park et al., 2007; Jiménez et al., 2011). Introduction of *Ca*. Nitrotoga sp. in dynamic WWTPs or other engineered systems may be beneficial due to their potential ability to maintain nitrite oxidation over a broader pH range.

### Impact of Temperature on *Ca*. Nitrotoga sp. CP45 Nitrite Oxidation

*Ca*. Nitrotoga sp. CP45 oxidized nitrite when incubated at temperatures ranging from 4 to 28°C (Figures 2, 3 and Supplementary Table 1), suggesting physiological temperature adaptability enabling maintenance of nitrite oxidation over the typical *in situ* temperature variability within the river year-round (Supplementary Figure 2). Nitrite oxidation rates were highest at 25°C (256 μM NO_2_^–^ day^–1^). When incubated at 23 and 28°C, *Ca*. Nitrotoga sp. CP45 oxidized nitrite at similar rates to the optimum. Activity completely halted at 30°C.

CP45 nitrite oxidation at ambient temperatures is consistent with physiological characteristics of other *Ca*. Nitrotoga sp. The reported optimum temperature for described *Ca.* Nitrotoga species ranged from 10 to 28°C across a wide range of inoculum sources: 10°C for *Ca.* Nitrotoga arctica cultivated from Siberian Arctic permafrost soils (Alawi et al., 2007; Nowka et al., 2015); 16°C for *Ca.* Nitrotoga AM1 and 23°C for AM1P cultivated from marine sediments (Ishii et al., 2017, 2020); 17–22°C for *Ca.* Nitrotoga BS enriched from activated sludge (Wegen et al., 2019); 22°C for *Ca.* Nitrotoga HW29 cultivated from a recirculating aquaculture system (Hüpeden et al., 2016); and 24°C –28°C for *Ca.* Nitrotoga fabula strain KNB cultivated from activated sludge (Kitzinger et al., 2018). Our previous analyses described *Ca.* Nitrotoga-like sequences in samples with reported temperatures ranging from 0 to 33°C across many sample types (based on searches of the NCBI Sequence Read Archive with associated temperature metadata); however, *Ca.* Nitrotoga-like sequences were only identified in freshwater samples with reported temperatures < 15°C (Boddicker and Mosier, 2018). Our findings suggest that nitrite oxidation by *Ca*. Nitrotoga sp. in freshwater systems may occur over a broader range of temperatures.

*Ca*. Nitrotoga sp. CP45 oxidized nitrite at the lowest temperature tested (4°C) but activity was much slower than at optimum temperatures (Figure 3). The ability to continue nitrite oxidation at cold temperatures is useful for maintenance of ecological health in local rivers given the seasonal variation and consequential temperature fluctuation that occur across the year. At the river enrichment site, water temperature averaged 12–15°C over the year but dropped below 5°C from late November to the middle of March (Supplementary Figure 2). We analyzed the CP45 genome and identified several proteins that may be involved in counteracting the harmful effects of cold temperatures including cold-shock proteins such as CspA, oxidative stress response proteins such as catalase and superoxide dismutase, and proteins for the production of extracellular polysaccharides (EPS) such as alginate (Smirnova et al., 2001; Margesin and Miteva, 2011; Keto-Timonen et al., 2016). Though nitrite oxidation rates are likely quite low in the winter months, *Ca*. Nitrotoga sp. CP45 may be able to outcompete more temperature-sensitive NOB in the system. For instance, *Ca.* Nitrotoga were previously shown to outcompete *Nitrospira* and *Nitrobacter* spp. at lower temperatures (Alawi et al., 2009).

### Impact of Antibiotic Exposure on *Ca*. Nitrotoga sp. CP45 Nitrite Oxidation

**FIGURE 4:**
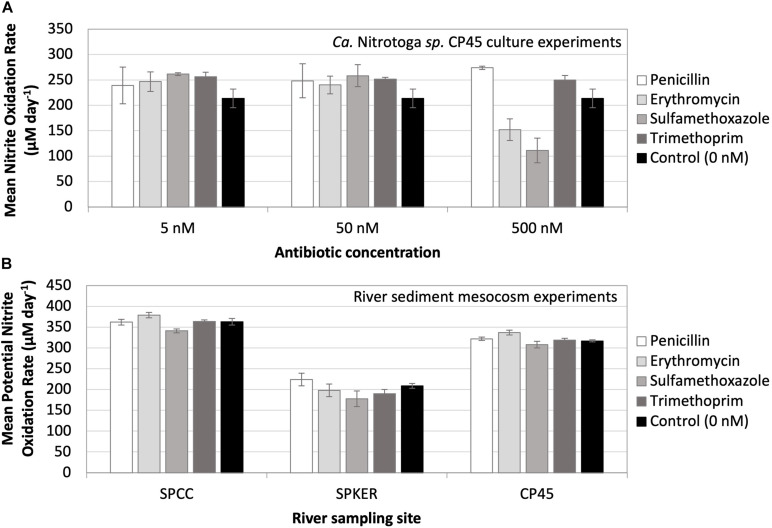
**(A)** Mean nitrite oxidation rates (μM day^– 1^) by *Ca*. Nitrotoga sp. CP45 in the presence of 5, 50, and 500 nM concentrations of four different antibiotics (penicillin, erythromycin, sulfamethoxazole, and trimethoprim). Controls contained no antibiotics. **(B)** Mean potential nitrite oxidation rates (μM day^– 1^) from river mesocosm experiments where sediments from three river sites (SPCC, SPKER, and CP45) were exposed to four antibiotics (penicillin, erythromycin, sulfamethoxazole, and trimethoprim) at a concentration of 500 nM. Antibiotic-free sediments were utilized to represent inherent, endogenous microbial nitrite oxidation activity at each site. Each symbol represents the average of triplicate cultures or sediment incubations grown under each condition with standard deviation error bars.

Antibiotics have emerged as a contaminant of concern in riverine systems influenced by human activity (Kolpin et al., 2002); however, their ecological effects have been poorly investigated despite the fact that many antibiotics possess broad-spectrum mechanisms of action that may negatively impact non-target organisms naturally present in the environment (Grenni et al., 2018). Here, we exposed *Ca*. Nitrotoga sp. CP45 cultures to four antibiotics commonly found in contaminated rivers (penicillin, erythromycin, sulfamethoxazole, and trimethoprim) at three concentrations each (5, 50, and 500 nM). Results indicated that in the presence of 5–50 nM of each antibiotic, *Ca*. Nitrotoga sp. CP45 oxidized nitrite at a slightly increased rate (though not significantly different) compared to the control culture with no antibiotics present (Figure 4). The 500 nM treatment had a more variable effect across the different antibiotics. *Ca*. Nitrotoga sp. CP45 had slightly increased nitrite oxidation rates with 500 nM penicillin and trimethoprim exposure (no significant difference). Rates decreased with 500 nM erythromycin (*P* = 0.019) and sulfamethoxazole (*P* = 0.004) exposure (Figure 4).

Overall, these findings show that *Ca*. Nitrotoga sp. CP45 is capable of maintaining nitrite oxidation in the presence of antibiotics. Increased nitrite oxidation rates in the presence of antibiotics (though not necessarily significantly different from the control) could be the result of decreased competition with other bacteria in the enrichment culture, increased resource availability from dying antibiotic-susceptible cells (e.g., release of nutrient rich macromolecules), direct nutrient supply from the antibiotic chemicals (e.g., carbon, nitrogen, and sulfur in the case of penicillin and sulfamethoxazole), or altered metabolism (e.g., heterotrophy versus autotrophy). Decreased nitrite oxidation rates at higher antibiotic concentrations (500 nM erythromycin and sulfamethoxazole) are likely indicative of physiological stress. The lowest antibiotic concentrations tested here were about twice as high as the *in situ* antibiotic concentrations previously measured in the river water column (for sulfamethoxazole and trimethoprim; Bai et al., 2018). This suggests that *Ca*. Nitrotoga sp. CP45 could likely maintain nitrite oxidation at much higher levels of antibiotic pollution in the river.

We extended these culture studies to evaluate the impact of antibiotics on nitrite oxidation by naturally occurring microbes in sediments at three sites with varying land-use patterns in the South Platte River Basin (Figure 4): SPCC, influenced primarily by WWTP effluent; CP45, influenced primarily by runoff from animal feeding operations (AFO); and SPKER with a mixed land-use of urban and agriculture. Each of these sources of runoff can be associated with antibiotic pollution that varies by land use (e.g., antibiotics found in human versus animal waste) (Barnes et al., 2002; Scribner et al., 2003; Koike et al., 2010; Roose-Amsaleg and Laverman, 2015). Overall, we observed rapid potential rates of nitrite oxidation in river sediments at each site. Fastest overall potential rates were observed at WWTP- and AFO-dominated sites (SPCC and CP45), which may be attributed to differences in microbial community composition, contaminant history, sediment structure, or abiotic factors (e.g., temperature). Potential rates of nitrite oxidation were similar to controls when incubated in the presence of elevated antibiotics. Quantitative PCR estimates of NOB abundance from the sediment inoculum at each site revealed that *Ca.* Nitrotoga 16S rRNA gene copies (∼1.4 × 10^5^ copies per gram of sediment) were more abundant than *Nitrospira* 16S rRNA gene copies at each site (∼7.8 × 10^4^ copies per gram of sediment) (*Nitrobacter* genes were not amplifiable at any site). Mesocosm experiments using the river water column as inoculum (without added sediment) showed essentially no NOB amplification by qPCR and no nitrite oxidation with or without antibiotic amendments. The complex nature of the sediment microbial community and metabolite profile may influence the nitrogen and carbon pools in these river mesocosm experiments, so rates should be interpreted as the “potential” for nitrite oxidation under the given conditions (with the possibility of other biotic and abiotic influences). Further research is needed to determine the activity of specific community members upon antibiotic exposure.

Limited studies have evaluated the impact of antibiotics on nitrogen cycling. Studies focusing on the effect of antibiotics on nitrification in activated sludge revealed that benzylpenicillin exposure resulted in no effect on the rate of nitrate production, while erythromycin demonstrated an inhibition in ammonification, nitritation, and nitratation (Gomez et al., 1996; Alighardashi et al., 2009; Katipoglu-Yazan et al., 2013). Additionally, nitrite oxidation in a long-term laboratory-scale treatment plant was inhibited when exposed to a mixture of ciprofloxacin, gentamicin, sulfamethoxazole/trimethoprim, and vancomycin resulting in no nitrate formation (Schmidt et al., 2012). Variability in the overall effect of antibiotics in different systems is likely a result of differences in tolerance of the underlying microbial community. Also, the complexity of each individual antibiotic class (e.g., absorption, pharmacokinetics, metabolite formation, degradation, and sediment adsorption) affects the bioavailability of the antibiotic once it enters the waterway (Luo et al., 2011).

Here, the uniform rates of potential nitrite oxidation by endogenous sediment communities across the three sampling sites and in culture would suggest mechanisms for tolerating the effects of antibiotics. NOB are Gram-negative bacteria that characteristically possess an additional outer membrane that can serve as protection against large antibacterial compounds that cannot penetrate the cell wall and enter the cytoplasm. Furthermore, many NOB can produce EPS (including *Ca*. Nitrotoga sp. CP45 based on genomic predictions; see above) that facilitate biofilm formation (Hüpeden et al., 2016), which may protect interior NOB from antibiotics at the surface of the biofilm. Acquired antibiotic resistance can occur by adaptive gene mutation in the presence of a stressor (such as antibiotics) or through the process of genetic exchange mechanisms (e.g., transformation, transduction, or conjugation) (Rosenblatt-Farrell, 2009). Previous research identified antibiotic pollution (Yang and Carlson, 2003; Pei et al., 2006; Pruden et al., 2006; Kim and Carlson, 2007; Bai et al., 2018) and antibiotic resistance genes (Storteboom et al., 2010a,b) in the South Platte River Basin. The NOB studied here likely also have developed some mechanism of antibiotic resistance in order to maintain nitrite oxidation during antibiotic exposure. The *Ca*. Nitrotoga sp. CP45 genome was predicted to encode a broad-spectrum antibiotic efflux pump that may confer resistance to all four antibiotics tested here (Boddicker and Mosier, 2018; based on searches against the CARD database, Alcock et al., 2020). Expanded annotation analyses here indicated that the *Ca*. Nitrotoga sp. CP45 genome was predicted to also encode a MdtABC multidrug exporter system (Nagakubo et al., 2002), as well as several additional putative multidrug efflux proteins identified by manual annotation. Riverine NOB capable of nitrite oxidation in the mesocosm experiments likely exhibited similar resistance mechanisms. These antibiotic-resistant NOB may spread their resistance genes to other microbes in the river *via* mechanisms of genetic exchange. Future efforts should evaluate temporal trends in antibiotic exposure, gene expression of NOB under antibiotic stress, and the effects of contaminant mixtures common within river systems.

### Influence of Community Members on Nitrite Oxidation by *Ca.* Nitrotoga sp. CP45

*Ca*. Nitrotoga sp. CP45 was grown in enrichment cultures, so other community members also likely influence their physiology. At the time of genomic sequencing, other organisms (besides *Ca*. Nitrotoga sp. CP45) in the CP45 enrichment culture were identified as p__Bacteroidetes, o__Burkholderiales, o__Rhizobiales, and o__Pseudomonadales by checkM (Parks et al., 2015); *Ca*. Nitrotoga sp. CP45 was enriched over time to > 65% abundance (Boddicker and Mosier, 2018). No other organisms capable of nitrite oxidation or ammonia oxidation were present based on evaluation of 16S rRNA gene sequences and protein coding genes sequenced in the culture metagenome. *Ca*. Nitrotoga sp. CP45 may utilize metabolites and/or cofactors produced by heterotrophs within the culture to compensate for incomplete biosynthetic pathways within their own genome and/or to reduce biosynthetic costs (e.g., Daims et al., 2016; Ngugi et al., 2016). Likewise, *Ca.* Nitrotoga sp. CP45 metabolism may influence other community members (e.g., production of organic carbon, pH impacts). As biomass builds up toward the end of nitrite oxidation, organic carbon likely results in a dynamic exchange of metabolites and cross-feeding among the community members, as well as other chemical changes (e.g., oxygen and pH). When biomass is available, some community members may be capable of aerobic denitrification using organic carbon as an electron donor (inorganic electron donors such as hydrogen or sulfur can be used by some organisms under certain circumstances) and nitrite/nitrate as an electron acceptor for conversion to nitrogen gases. This is unlikely to occur in the early phases of *Ca*. Nitrotoga sp. CP45 growth since the culture conditions contained nitrite as the only known electron donor and oxygen as the only electron acceptor. Adjustments to culture conditions (e.g., low or high pH) may alter the activity of the other non-NOB community members depending on their own physiology (range and optima for each parameter tested). Abiotic sources and sinks may also influence the community dynamics. Nonetheless, our data suggest that *Ca*. Nitrotoga sp. CP45 oxidizes nitrite to nitrate (with corresponding increases in cell counts) during the exponential growth phase, so the nitrite data presented likely reflect metabolic processes specific to these cells. In some ways, these complex community relationships within the culture hint at the reality of *Ca*. Nitrotoga sp. CP45 living in a dynamic ecosystem with fluctuating biological, chemical, and physical factors.

## Conclusion

*Ca.* Nitrotoga has emerged as a genus of versatile organisms with a diverse metabolic potential. Physiological tests showed that nitrite oxidation can occur in darkness, across all pH values tested (pH 5.0–8.0), at temperatures ranging from 4 to 28°C, and in the presence of antibiotics. The ability of *Ca*. Nitrotoga sp. CP45 to oxidize nitrite across a variety of conditions indicates that the environmental range of *Ca.* Nitrotoga may be greater than initially estimated. The physiological versatility of *Ca.* Nitrotoga may be ecologically valuable in natural and engineered environments that rely on the function of NOB to mitigate nitrite toxicity. By recognizing potential constraining variables that may limit nitrite oxidation, future management decisions may be guided to best manage elevated levels of nitrogen in contaminated systems that rely on the activity of NOB communities for mitigation. Understanding the influence of key microorganisms, such as NOB, strengthens the predictive power required to recognize and manage nitrogen levels that, if allowed to intensify, may otherwise result in a cascade of environmental and human health complications.

## Data Availability Statement

The original contributions presented in the study are included in the article/Supplementary Material, further inquiries can be directed to the corresponding author/s.

## Author Contributions

ML and AM conceived and designed the experiments. ML, CW, and AM contributed to sampling, site information, and chemistry. ML, AB, MK, OB, and AM performed the experiments. ML, AB, and AM analyzed the data. ML, AB, MK, OB, CW, and AM contributed to the manuscript. All authors contributed to the article and approved the submitted version.

## Conflict of Interest

The authors declare that the research was conducted in the absence of any commercial or financial relationships that could be construed as a potential conflict of interest.

## Publisher’s Note

All claims expressed in this article are solely those of the authors and do not necessarily represent those of their affiliated organizations, or those of the publisher, the editors and the reviewers. Any product that may be evaluated in this article, or claim that may be made by its manufacturer, is not guaranteed or endorsed by the publisher.

## Supplementary Material

Supplementary Figure 1 The concentration of **(A)** ammonia as nitrogen (μM NH_3_-N) and **(B)** nitrate and nitrite as nitrogen (μM NO_3_^–^ + NO_2_^–^-N) in the experimental dataset obtained by the Northern Water Conservation District at the nearby site approximately 2 miles away on the same river.

Supplementary Figure 2 Water column data measured at the CP45 site (gray symbols) or obtained by the Northern Water Conservation District at the nearby site approximately 2 miles away (black symbols) for **(A)** pH, **(B)** turbidity, and **(C)** temperature. **(D)** Water temperature at 15-min intervals around the clock for the 12-month time period from a Northern Water Conservation District sampling site approximately 9 miles away from the experimental sampling site.

Supplementary Table 1 Mean nitrite oxidation rates (μM day^–1^) at varying treatment conditions, with accompanying standard deviation values of replicate cultures. Data should be compared within an experiment, but not necessarily between experiments (see section “Materials and Methods”).

## References

[B1] Alawi M. Lipski A. Sanders T. Pfeiffer E. M. Spieck E. (2007). Cultivation of a novel cold-adapted nitrite oxidizing betaproteobacterium from the Siberian Arctic. *ISME J.* 1 256–264. 10.1038/ismej.2007.34 18062041

[B2] Alawi M. Off S. Kaya M. Spieck E. (2009). Temperature influences the population structure of nitrite-oxidizing bacteria in activated sludge. *Environ. Microbiol. Rep.* 1 184–190. 10.1111/j.1758-2229.2009.00029.x 23765792

[B3] Alcock B. P. Raphenya A. R. Lau T. T. Y. Tsang K. K. Bouchard M. Edalatmand A. (2020). CARD 2020: antibiotic resistome surveillance with the comprehensive antibiotic resistance database. *Nucleic Acids Res.* 48 D517–D525. 31665441 10.1093/nar/gkz935 PMC7145624

[B4] Alighardashi A. Pandolfi D. Potier O. Pons M. N. (2009). Acute sensitivity of activated sludge bacteria to erythromycin. *J. Hazard. Mater.* 172 685–692. 10.1016/j.jhazmat.2009.07.051 19674840

[B5] Altmann D. Stief P. Amann R. Beer D. D. Schramm A. (2003). *In situ* distribution and activity of nitrifying bacteria in freshwater sediment. *Environ. Microbiol.* 5 798–803. 10.1046/j.1469-2920.2003.00469.x 12919415

[B6] Altschul S. Gish W. Miller W. Myers E. Lipman D. (1990). Basic local alignment search tool. *J. Mol. Biol.* 215 403–410. 2231712 10.1016/S0022-2836(05)80360-2

[B7] American Public Health Association, American Water Works Association, and Water Environment Federation (2017). *Standard Methods for the Examination of Water and Wastewater, 23rd Edition 4500-NO_2_^–^ B. Colorimetric Method.* Washington, D.C: American Public Health Association, American Water Works Association, Water Environment Federation.

[B8] Austin ÅN. Hansen J. P. Donadi S. Eklöf J. S. (2017). Relationships between aquatic vegetation and water turbidity: a field survey across seasons and spatial scales. *PLoS One* 12:e0181419. 10.1371/journal.pone.0181419 28854185 PMC5576641

[B9] Bai X. Lutz A. Carroll R. Keteles K. Dahlin K. Murphy M. (2018). Occurrence, distribution, and seasonality of emerging contaminants in urban watersheds. *Chemosphere* 200 133–142. 10.1016/j.chemosphere.2018.02.106 29477762 PMC6705126

[B10] Baker-Austin C. Dopson M. (2007). Life in acid: pH homeostasis in acidophiles. *Trends Microbiol.* 15 165–171. 10.1016/j.tim.2007.02.005 17331729

[B11] Barnes K. K. Kolpin D. W. Meyer M. T. Thurman E. M. Furlong E. T. Zaugg S. D. (2002). *Water-Quality Data for Pharmaceuticals, Hormones, and Other Organic Wastewater Contaminants in U.S. Streams, 1999-2000 (2002-94).* Reston, VA: U.S. Geological Survey. 10.1021/es011055j 11944670

[B12] Blackburne R. Vadivelu V. M. Yuan Z. Keller J. (2007). Kinetic characterisation of an enriched *Nitrospira* culture with comparison to *Nitrobacter*. *Water Res.* 41 3033–3042. 10.1016/j.watres.2007.01.043 17553540

[B13] Blum J.-M. Su Q. Ma Y. Valverde-Pérez B. Domingo-Félez C. Jensen M. M. (2018). The pH dependency of N-converting enzymatic processes, pathways and microbes: effect on net N2O production. *Environ. Microbiol.* 20 1623–1640. 10.1111/1462-2920.14063 29411510

[B14] Blum M. Chang H. Chuguransky S. Grego T. Kandasaamy S. Mitchell A. (2021). The InterPro protein families and domains database: 20 years on. *Nucleic Acids Res.* 29 D344–D354. 10.1093/nar/gkaa977 33156333 PMC7778928

[B15] Bock E. (1970). Untersuchungen über die wechselwirkung zwischen licht und chemosynthese am beispiel von nitrobacter winogradskyi. *Arch. Mikrobiol.* 70 217–239. 10.1007/bf00407712 5437117

[B16] Bock E. Wagner M. (2013). “Oxidation of inorganic nitrogen compounds as an energy source,” in *The Prokaryotes*, eds Dworkin M. Falkow S. Rosenberg E. Schleifer K. H. Stackebrandt E. (New York, NY: Springer), 83–118. 10.1007/978-3-642-30141-4_64

[B17] Boddicker A. M. Mosier A. C. (2018). Genomic profiling of four cultivated *Candidatus* Nitrotoga spp. predicts broad metabolic potential and environmental distribution. *ISME J.* 12 2864–2882. 10.1038/s41396-018-0240-8 30050164 PMC6246548

[B18] Booth I. (1985). Regulation of cytoplasmic pH in Bacteria. *Microbiol. Rev.* 49 359–378. 10.1128/mmbr.49.4.359-378.1985 3912654 PMC373043

[B19] Cébron A. Garnier J. (2005). *Nitrobacter* and *Nitrospira* genera as representatives of nitrite-oxidizing bacteria: detection, quantification and growth along the lower Seine River (France). *Water Res.* 39 4979–4992. 10.1016/j.watres.2005.10.006 16303163

[B20] Chen Y. Wu L. Boden R. Hillebrand A. Kumaresan D. Moussard H. (2009). Life without light: microbial diversity and evidence of sulfur- and ammonium-based chemolithotrophy in Movile Cave. *ISME J.* 3 1093–1104. 10.1038/ismej.2009.57 19474813

[B21] Christner B. C. Priscu J. C. Achberger A. M. Barbante C. Carter S. P. Christianson K. (2014). A microbial ecosystem beneath the West Antarctic ice sheet. *Nature* 512 310–313. 10.1038/nature13667 25143114

[B22] Daims H. Lucker S. Wagner M. (2016). A new perspective on microbes formerly known as nitrite-oxidizing bacteria. *Trends Microbiol.* 24 699–712. 10.1016/j.tim.2016.05.004 27283264 PMC6884419

[B23] Dennehy K. F. Litke D. W. Tate C. M. Qi S. L. McMahon P. B. Bruce B. W. (1998). *Water Quality in the South Platte River Basin, Colorado, Nebraska, and Wyoming*. 1992–1995.

[B24] Fan L. Song C. Meng S. Qiu L. Zheng Y. Wu W. (2016). Spatial distribution of planktonic bacterial and archaeal communities in the upper section of the tidal reach in Yangtze River. *Sci. Rep.* 6:39147. 10.1038/srep39147 27966673 PMC5155431

[B25] Féray C. Volat B. Degrange V. Clays-Josserand A. Montuelle B. (1999). Assessment of three methods for detection and quantification of nitrite-oxidizing bacteria and *Nitrobacter* in freshwater sediments (MPN-PCR, MPN-Griess, Immunofluorescence). *Microb. Ecol.* 37 208–217. 10.1007/s002489900144 10227878

[B26] Gomez J. Mendez R. Lema J. M. (1996). The effect of antibiotics on nitrification processes. *Appl. Biochem. Biotechnol.* 57 869–876. 10.1007/bf02941767 8669922

[B27] Graham D. W. Knapp C. W. Van Vleck E. S. Bloor K. Lane T. B. Graham C. E. (2007). Experimental demonstration of chaotic instability in biological nitrification. *The ISME Journal* 1 385–393. 10.1038/ismej.2007.45 18043658

[B28] Grenni P. Ancona V. Barra Caracciolo A. (2018). Ecological effects of antibiotics on natural ecosystems: A review. *Microchem. J.* 136 25–39. 10.1016/j.microc.2017.02.006

[B29] Griess-Romijn van Eck E. (1966). *Physiological and Chemical Tests for Drinking Water.* Rijswijk: Nederlands Normalisatie Instituut 504 1056.

[B30] Grunditz C. Dalhammar G. (2001). Development of nitrification inhibition assays using pure cultures of *Nitrosomonas* and *Nitrobacter*. *Water Res.* 35 433–440. 10.1016/s0043-1354(00)00312-2 11228996

[B31] Guerrero M. Jones R. (1996). Photoinhibition of marine nitrifying bacteria. I. Wavelength-dependent response. *Mar. Ecol. Progr. Ser.* 141 183–192. 10.3354/meps141183

[B32] Hankinson T. R. Schmidt E. L. (1988). An acidophilic and a neutrophilic *Nitrobacter* strain isolated from the numerically predominant nitrite-oxidizing population of an acid forest soil. *Appl. Environ. Microbiol.* 54 1536–1540. 10.1128/aem.54.6.1536-1540.1988 16347664 PMC202692

[B33] Hooper A. B. Terry K. R. (1974). Photoinactivation of ammonia oxidation in *Nitrosomonas*. *J. Bacteriol.* 119 899–906. 10.1128/jb.119.3.899-906.1974 4369012 PMC245697

[B34] Hovanec T. A. Taylor L. T. Blakis A. Delong E. F. (1998). *Nitrospira*-like bacteria associated with nitrite oxidation in freshwater aquaria. *Appl. Environ. Microbiol.* 64 258–264. 10.1128/aem.64.1.258-264.1998 16349486 PMC124703

[B35] Huerta-Cepas J. Forslund K. Coelho L. P. Szklarczyk D. Jensen L. J. von Mering C. (2017). Fast genome-wide functional annotation through orthology assignment by eggNOG-Mapper. *Mol. Biol. Evol.* 34 2115–2122. 10.1093/molbev/msx148 28460117 PMC5850834

[B36] Huerta-Cepas J. Szklarczyk D. Heller D. Hernandez-Plaza A. Forslund S. K. Cook H. (2019). eggNOG 5.0: a hierarchical, functionally and phylogenetically annotated orthology resource based on 5090 organisms and 2502 viruses. *Nucleic Acids Res.* 47 D309–D314. 10.1093/nar/gky1085 30418610 PMC6324079

[B37] Hüpeden J. Wegen S. Off S. Lücker S. Bedarf Y. Daims H. (2016). Relative abundance of *Nitrotoga* spp. in a biofilter of a cold-freshwater aquaculture plant appears to be stimulated by slightly acidic pH. *Appl. Environ. Microbiol.* 82 1838–1845. 10.1128/aem.03163-15 26746710 PMC4784051

[B38] Ishii K. Fujitani H. Sekiguchi Y. Tsuneda S. (2020). Physiological and genomic characterization of a new ‘*Candidatus* Nitrotoga’ isolate. *Environ. Microbiol.* 22 2365–2382. 10.1111/1462-2920.15015 32285573

[B39] Ishii K. Fujitani H. Soh K. Nakagawa T. Takahashi R. Tsuneda S. (2017). Enrichment and physiological characterization of a cold-adapted nitrite-oxidizing *Nitrotoga* sp. from an eelgrass sediment. *Appl. Environ. Microbiol.* 83:e00549-17. 10.1128/aem.00549-17 28500038 PMC5494630

[B40] Jiménez E. Giménez J. Ruano M. Ferrer J. Serralta J. (2011). Effect of pH and nitrite concentration on nitrite oxidation rate. *Bioresour. Technol.* 102 8741–8747. 10.1016/j.biortech.2011.07.092 21843934

[B41] Kaplan D. Wilhelm R. Abeliovich A. (2000). Interdependent environmental factors controlling nitrification in waters. *Water Sci. Technol.* 42 167–172. 10.2166/wst.2000.0309

[B42] Katipoglu-Yazan T. Pala-Ozkok I. Ubay-Cokgor E. Orhon D. (2013). Acute impact of erythromycin and tetracycline on the kinetics of nitrification and organic carbon removal in mixed microbial culture. *Bioresour. Technol.* 144 410–419. 10.1016/j.biortech.2013.06.121 23892149

[B43] Keto-Timonen R. Hietala N. Palonen E. Hakakorpi A. Lindstrom M. Korkeala H. (2016). Cold shock proteins: a minireview with special emphasis on Csp-family of enteropathogenic *Yersinia*. *Front. Microbiol.* 7:1151. 10.3389/fmicb.2016.01151 27499753 PMC4956666

[B44] Kim S. C. Carlson K. (2007). Temporal and spatial trends in the occurrence of human and veterinary antibiotics in aqueous and river sediment matrices. *Environ. Sci. Technol.* 41 50–57. 10.1021/es060737 17265926

[B45] Kitzinger K. Koch H. Lücker S. Sedlacek C. J. Herbold C. Schwarz J. (2018). Characterization of the first “*Candidatus* Nitrotoga” isolate reveals metabolic versatility and separate evolution of widespread nitrite-oxidizing bacteria. *mBio* 9:e01186-18. 10.1128/mBio.01186-18 29991589 PMC6050957

[B46] Koike S. Aminov R. I. Yannarell A. C. Gans H. D. Krapac I. G. Chee-Sanford J. C. (2010). Molecular ecology of macrolide–lincosamide–streptogramin B methylases in waste lagoons and subsurface waters associated with swine production. *Microb. Ecol.* 59 487–498. 10.1007/s00248-009-9610-0 19924466

[B47] Kolpin D. W. Furlong E. T. Meyer M. T. Thurman E. M. Zaugg S. D. Barber L. B. (2002). Pharmaceuticals, hormones, and other organic wastewater contaminants in U.S. streams, 1999-2000: a national reconnaissance. *Environ. Sci. Technol.* 36 1202–1211. 10.1021/es011055j 11944670

[B48] Krulwich T. A. Sachs G. Padan E. (2011). Molecular aspects of bacterial pH sensing and homeostasis. *Nat. Rev. Microbiol.* 9 330–343. 10.1038/nrmicro2549 21464825 PMC3247762

[B49] Lantz M. (2019). *Impacts of Environmental Change on Freshwater Nitrite Oxidation*. University of Colorado Denver.

[B50] Lehtovirta-Morley L. E. Sayavedra-Soto L. A. Gallois N. Schouten S. Stein L. Y. Prosser J. I. (2016). Identifying potential mechanisms enabling acidophily in the ammonia-oxidizing archaeon “*Candidatus* Nitrosotalea devanaterra”. *Appl. Environ. Microbiol.* 82 2608–2619. 10.1128/AEM.04031-15 26896134 PMC4836417

[B51] Li D. Qi R. Yang M. Zhang Y. Yu T. (2011). Bacterial community characteristics under long-term antibiotic selection pressures. *Water Res.* 45 6063–6073. 10.1016/j.watres.2011.09.002 21937072

[B52] Liu B. Zheng D. D. Jin Q. Chen L. H. Yang J. (2019). VFDB 2019: a comparative pathogenomic platform with an interactive web interface. *Nucleic Acids Res.* 47 D687–D692. 30395255 10.1093/nar/gky1080 PMC6324032

[B53] Lücker S. Schwarz J. Gruber-Dorninger C. Spieck E. Wagner M. Daims H. (2014). *Nitrotoga*-like bacteria are previously unrecognized key nitrite oxidizers in full-scale wastewater treatment plants. *ISME J.* 9 708–720. 10.1038/ismej.2014.158 25180967 PMC4270731

[B54] Luo Y. Xu L. Rysz M. Wang Y. Zhang H. Alvarez P. J. J. (2011). Occurrence and transport of tetracycline, sulfonamide, quinolone, and macrolide antibiotics in the haihe river basin, China. *Environ. Sci. Technol.* 45 1827–1833. 10.1021/es104009s 21309601

[B55] Margesin R. Miteva V. (2011). Diversity and ecology of psychrophilic microorganisms. *Res. Microbiol.* 162 346–361. 10.1016/j.resmic.2010.12.004 21187146

[B56] Merbt S. N. Stahl D. A. Casamayor E. O. Martí E. Nicol G. W. Prosser J. I. (2012). Differential photoinhibition of bacterial and archaeal ammonia oxidation. *FEMS Microbiol. Lett.* 327 41–46. 10.1111/j.1574-6968.2011.02457.x 22093004

[B57] Miranda K. M. Espey M. G. Wink D. A. (2001). A rapid, simple spectrophotometric method for simultaneous detection of nitrate and nitrite. *Nitric Oxide Biol. Chem.* 5 62–71. 10.1006/niox.2000.0319 11178938

[B58] Nagakubo S. Nishino K. Hirata T. Yamaguchi A. (2002). The putative response regulator BaeR stimulates multidrug resistance of *Escherichia coli* via a novel multidrug exporter system, MdtABC. *J. Bacteriol.* 184 4161–4167. 10.1128/jb.184.15.4161-4167.2002 12107133 PMC135206

[B59] Ngugi D. K. Blom J. Stepanauskas R. Stingl U. (2016). Diversification and niche adaptations of *Nitrospina*-like bacteria in the polyextreme interfaces of Red Sea brines. *ISME J.* 10 1383–1399. 10.1038/ismej.2015.214 26657763 PMC5029188

[B60] Northern Water Conservancy District (2016). East Slope Temperature Data. Available online at: https://www.northernwater.org/WaterQuality/EastSlopeWaterTemperatureData.aspx; https://www.northernwater.org/DynData/WQDataMain.aspx

[B61] Nowka B. Daims H. Spieck E. (2015). Comparison of oxidation kinetics of nitrite-oxidizing bacteria: nitrite availability as a key factor in niche differentiation. *Appl. Environ. Microbiol.* 81, 745–753. 10.1128/AEM.02734-14 25398863 PMC4277589

[B62] Pal C. Bengtsson-Palme J. Rensing C. Kristiansson E. Larsson D. G. (2014). BacMet: antibacterial biocide and metal resistance genes database. *Nucleic Acids Res.* 42 D737–D743. 10.1093/nar/gkt1252 24304895 PMC3965030

[B63] Park S. Bae W. Chung J. Baek S.-C. (2007). Empirical model of the pH dependence of the maximum specific nitrification rate. *Process Biochem.* 42 1671–1676. 10.1016/j.procbio.2007.09.010

[B64] Parks D. H. Imelfort M. Skennerton C. T. Hugenholtz P. Tyson G. W. (2015). CheckM: assessing the quality of microbial genomes recovered from isolates, single cells, and metagenomes. *Genome Res.* 25 1043–1055. 10.1101/gr.186072.114 25977477 PMC4484387

[B65] Pei R. Kim S. C. Carlson K. H. Pruden A. (2006). Effect of river landscape on the sediment concentrations of antibiotics and corresponding antibiotic resistance genes (ARG). *Water Res.* 40 2427–2435. 10.1016/j.watres.2006.04.017 16753197

[B66] Philips S. Laanbroek H. J. Verstraete W. (2002). Origin, causes and effects of increased nitrite concentrations in aquatic environments. *Rev. Environ. Sci. Biotechnol.* 1 115–141. 10.1023/a:1020892826575

[B67] Prosser J. (1989). Autotrophic nitrification in bacteria. *Adv. Microb. Physiol.* 30 125–181. 10.1016/s0065-2911(08)60112-5 2700538

[B68] Pruden A. Pei R. Storteboom H. Carlson K. H. (2006). Antibiotic resistance genes as emerging contaminants: studies in Northern Colorado. *Environ. Sci. Technol.* 40 7445–7450. 10.1021/es060413l 17181002

[B69] Roose-Amsaleg C. Laverman A. M. (2015). Do antibiotics have environmental side-effects? Impact of snythetic antibiotics on biogeochemical processes. *Environ. Sci. Pollut. Res.* 23 4000–4012. 10.1007/s11356-015-4943-3 26150293

[B70] Rosenblatt-Farrell N. (2009). The landscape of antibiotic resistance. *Environ. Health Perspect.s* 117 A244–A250. 10.1289/ehp.117-a244 PMC2702430 19590668

[B71] Sachs G. Weeks D. Wen Y. Marcus E. Scott D. (2005). Acid accumulation by *Helicobacter pylori*. *Physiology* 20 429–438. 10.1152/physiol.00032.2005 16287992

[B72] Schmidt S. Winter J. Gallert C. (2012). Long-term effects of antibiotics on the elimination of chemical oxygen demand, nitrification, and viable bacteria in laboratory-scale wastewater treatment plants. *Arch. Environ. Contam. Toxicol.* 63 354–364. 10.1007/s00244-012-9773-4 22622431

[B73] Schreiber F. Wunderlin P. Udert K. M. Wells G. F. (2012). Nitric oxide and nitrous oxide turnover in natural and engineered microbial communities: biological pathways, chemical reactions, and novel technologies. *Front. Microbiol.* 3:372. 10.3389/fmicb.2012.00372 23109930 PMC3478589

[B74] Scribner E. A. Battaglin W. A. Dietze J. E. Thurman E. M. (2003). *Reconnaissance Data for Glyphosate, Other Selected Herbicides, their Degradation Products, and Antibiotics in 51 Streams in Nine Midwestern States, 2002 (2003-217).* Reston, VA: U.S. Geological Survey.

[B75] Smirnova G. Zakirova O. Oktyabrskii O. (2001). The role of antioxidant systems in the cold stress response of *Escherichia coli*. *Microbiology* 70 45–50.11338835

[B76] Sorokin D. Y. Lücker S. Vejmelkova D. Kostrikina N. A. Kleerebezem R. Rijpstra W. I. C. (2012). Nitrification expanded: discovery, physiology and genomics of a nitrite-oxidizing bacterium from the phylum Chloroflexi. *ISME J.* 6, 2245–2256. 10.1038/ismej.2012.70 22763649 PMC3504966

[B77] Spieck E. Spohn M. Wendt K. Bock E. Shively J. Frank J. (2019). Extremophilic nitriteoxidizing Chloroflexi from Yellowstone hot springs. *ISME J.* 14. 10.1038/s41396-019-0530-9 [Epub ahead of print]. 31624340 PMC6976673

[B78] Spikes J. D. Livingston R. (1969). The molecular biology of photodynamic action: sensitized photoautoxidations in biological systems. *Adv. Radiat. Biol.* 3 29–121. 10.1016/b978-1-4832-3122-8.50008-1

[B79] Storteboom H. Arabi M. Davis J. G. Crimi B. Pruden A. (2010a). Identification of antibiotic-resistance-gene molecular signatures suitable as tracers of pristine river, urban, and agricultural sources. *Environ. Sci. Technol.* 44 1947–1953. 10.1021/es902893f 20158229

[B80] Storteboom H. Arabi M. Davis J. G. Crimi B. Pruden A. (2010b). Tracking antibiotic resistance genes in the South Platte River Basin using molecular signatures of urban, agricultural, and pristine sources. *Environ. Sci. Technol.* 44 7397–7404. 10.1021/es101657s 20809616

[B81] Takahashi Y. Fujitani H. Hirono Y. Tago K. Wang Y. Hayatsu M. (2020). Enrichment of comammox and nitrite-oxidizing *Nitrospira* from acidic soils. *Front. Microbiol.* 11:1737. 10.3389/fmicb.2020.01737 32849373 PMC7396549

[B82] Tang G. Wang S. Lu D. Huang L. Li N. Luo L. (2017). Two-component regulatory system ActS/ActR is required for Sinorhizobium meliloti adaptation to oxidative stress. *Microbiol. Res.* 198 1–7. 10.1016/j.micres.2017.01.005 28285657

[B83] Tiwari R. Reeve W. Dilworth M. Glenn A. (1996). Acid tolerance in *Rhizobium meliloti* strain WSM419 involves a two-component sensor-regulator system. *Microbiology* 142 1693–1704. 10.1099/13500872-142-7-1693 8757734

[B84] Vanzella A. Guerrero M. Jones R. (1989). Effect of CO and light on ammonium and nitrite oxidation by chemolithotrophic bacteria. *Mar. Ecol. Progr. Ser.* 57 69–76. 10.3354/meps057069

[B85] Wegen S. Nowka B. Spieck E. (2019). Low temperature and neutral pH define “*Candidatus* Nitrotoga sp.” as a competitive nitrite oxidizer in coculture with *Nitrospira defluvii*. *Appl. Environ. Microbiol.* 85:e02569-18. 10.1128/aem.02569-18 30824434 PMC6495747

[B86] White C. P. Debry R. W. Lytle D. A. (2012). Microbial survey of a full-scale, biologically active filter for treatment of drinking water. *Appl. Environ. Microbiol.* 78 6390–6394. 10.1128/aem.00308-12 22752177 PMC3416602

[B87] Yang S. Carlson K. (2003). Evolution of antibiotic occurrence in a river through pristine, urban and agricultural landscapes. *Water Res.* 37 4645–4656. 10.1016/S0043-1354(03)00399-3 14568051

[B88] Yoshioka T. Saijo Y. (1984). Photoinhibition and recovery of NH_4_-oxidizing bacteria and NO_2_-oxidizing bacteria. *J. Gen. Appl. Microbiol.* 30 151–166. 10.2323/jgam.30.151

[B89] Zafiriou O. C. True M. B. (1979). Nitrite photolysis in seawater by sunlight. *Mar. Chem.* 8 9–32. 10.1016/0304-4203(79)90029-x

