# Corrigendum: Physiology of the Nitrite-Oxidizing Bacterium *Candidatus* Nitrotoga sp. CP45 Enriched From a Colorado River

**DOI:** 10.3389/fmicb.2021.801108

**Published:** 2021-11-12

**Authors:** Munira A. Lantz, Andrew M. Boddicker, Michael P. Kain, Owen M. C. Berg, Courtney D. Wham, Annika C. Mosier

**Affiliations:** Department of Integrative Biology, University of Colorado Denver, Denver, CO, United States

**Keywords:** nitrite-oxidizing bacteria, freshwater, nitrification, water quality, nitrotoga, antibiotics, physiology, temperature

In the original article, there was an error in the Acknowledgment section where Gracie RedShirt Tyon's important contributions were unintentionally missed.

A correction has been made to the **Acknowledgment**:

We thank Hannah Clark, Bhargavi Ramanathan, Nicklaus Deevers, Colin Beacom, Ashley Luntsford, Ashley Gonzales, Renee Kershaw, Anna Scopp, Sladjana Subotic, Brian Legvold, and Ben Wise for their assistance in field sampling, cultivation, and/or preliminary physiology experiments. We thank Alan Vajda, Kristen Keteles, and Karl Hermann for discussions about river contaminant chemistry. Portions of this manuscript were previously published as a part of University of Colorado Denver Ph.D. Dissertation submission (Lantz, 2019). “We honor and acknowledge that the samples used for the research presented here were collected from rivers that are from the traditional territories and ancestral homelands of the Cheyenne, Arapaho, and Ute Nations, as well as over 45 Indigenous Nations who called this area home. The confluence of the Platte and Cherry Creek Rivers was the epicenter for trade, information sharing, planning for the future, community, family and ally building, and conducting healing ceremonies. We recognize Indigenous peoples as the original inhabitants, stewards, and relatives of this land. Let us acknowledge the painful history of genocide and forced removal from this territory and pay our respect to the diverse Indigenous peoples still connected to this land. We thank all Tribal Nations and the ancestors of this place.” We thank Gracie RedShirt Tyon (Lakota, Director of American Indian Student Services, University of Colorado at Denver) for assistance in understanding the history of this region and for writing this land acknowledgment statement.

The authors apologize for this error and state that this does not change the scientific conclusions of the article in any way. The original article has been updated.

## Publisher's Note

All claims expressed in this article are solely those of the authors and do not necessarily represent those of their affiliated organizations, or those of the publisher, the editors and the reviewers. Any product that may be evaluated in this article, or claim that may be made by its manufacturer, is not guaranteed or endorsed by the publisher.
