# Supplementary material for: Physiology of the Nitrite-Oxidizing Bacterium Candidatus Nitrotoga sp. CP45 Enriched From a Colorado River
Source: Front Microbiol. 2021 Aug 16;12:709371. doi: 10.3389/fmicb.2021.709371 (PMC8415719; doi:10.3389/fmicb.2021.709371)
Supplement: Supplementary Figure 1 — The concentration of (A) ammonia as nitrogen (μM NH3-N) and (B) nitrate and nitrite as nitrogen (μM NO3– + NO2–-N) in the experimental dataset obtained by the Northern Water Conservation District at the nearby site approximately 2 miles away on the same river. [file Data_Sheet_1.pdf]

Physiology of the nitrite-oxidizing bacterium *Candidatus* Nitrotoga sp. CP45 enriched from a Colorado river

Munira A. Lantz, Andrew M. Boddicker, Michael P. Kain, Owen M. C. Berg, Courtney D. Wham, Annika C. Mosier

Supplemental Material

Supplemental Table S1. Mean nitrite oxidation rates (µM day^{-1}) at varying treatment conditions, with accompanying standard deviation values of replicate cultures. Data should be compared within an experiment, but not necessarily between experiments (see Methods).

**Treatment**	**Condition**	**Mean nitrite oxidation rate (µM day^{-1})**	**Standard Deviation**
**Light/ Dark**	Light	3	1
	Dark	194	5
**pH**	pH 5.0	41	11
	pH 5.7	102	10
	pH 6.0	144	9
	pH 7.0	162	5
	pH 8.0	162	2
**Temperature**	4˚C	44	0
	10˚C	90	3
	15˚C	153	5
	20˚C	185	11
	23˚C	225	26
	25˚C	256	3
	28˚C	196	9
	30˚C	0	1

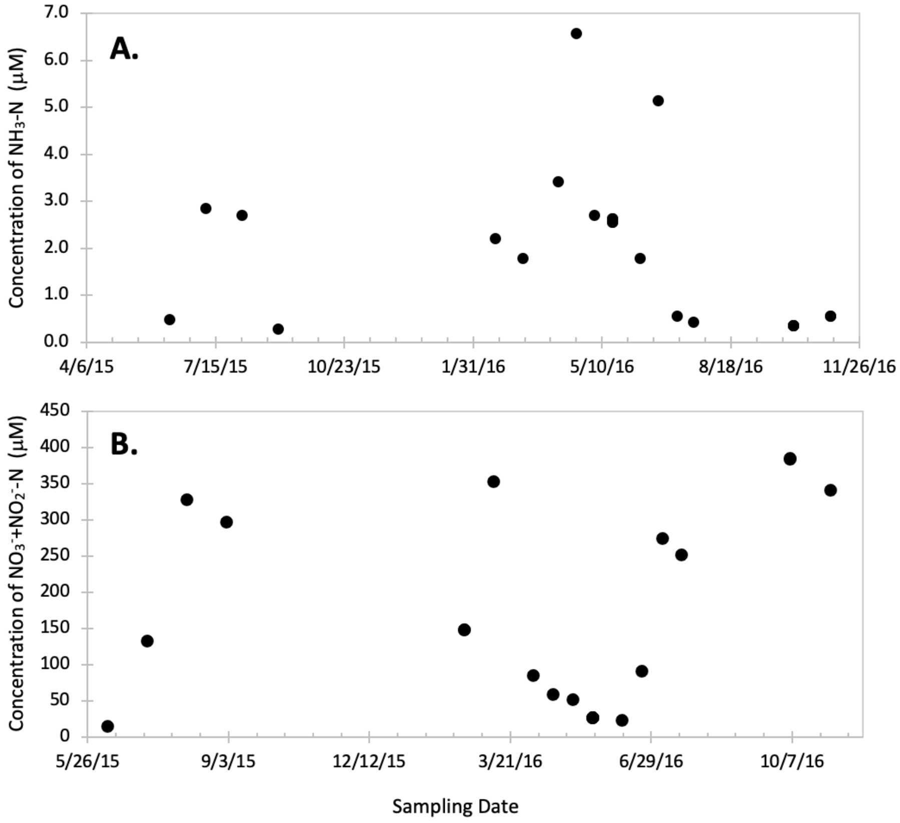

Supplemental Figure S1. The concentration of **(A)** ammonia as nitrogen (μM NH_3-N) and **(B)** nitrate and nitrite as nitrogen (μM NO_3^-+NO_2^--N) in the experimental dataset obtained by the Northern Water Conservation District at the nearby site approximately 2 miles away on the same river.

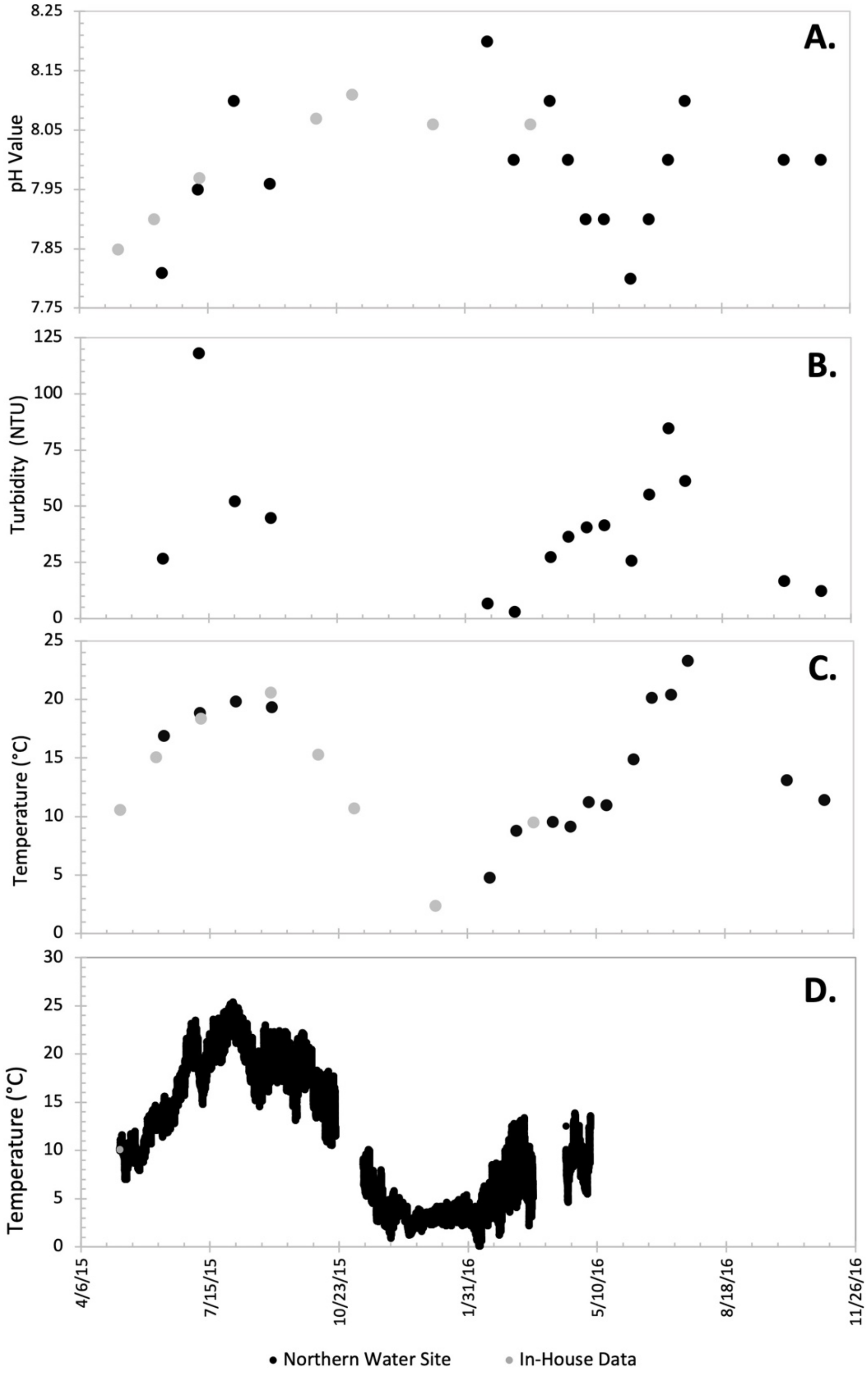

Supplemental Figure S2. Water column data measured at the CP45 site (grey symbols) or obtained by the Northern Water Conservation District at the nearby site approximately 2 miles away (black symbols) for **(A)** pH, **(B)** turbidity, and **(C)** temperature. (**D)** Water temperature at 15-minute intervals around the clock for the 12-month time period from a Northern Water Conservation District sampling site approximately 9 miles away from the experimental sampling site.
